# QI project: Improving the discharge advice from functional old age psychiatry wards for the monitoring of lithium and antipsychotic medication in the community

**DOI:** 10.1192/bjo.2021.551

**Published:** 2021-06-18

**Authors:** Amy Mathews, Nicole Needham

**Affiliations:** Royal Edinburgh Hospital

## Abstract

**Aims:**

NICE guidelines and Maudsley prescribing guidelines both stipulate that patients over the age of 65 prescribed lithium or antipsychotic medication should have their bloods and physical parameters monitored regularly. There is currently no provision from the community mental health teams in Edinburgh to provide this monitoring, which falls to the patients GP. Following an initial data collection, it was found that there was no monitoring advice being provided on immediate discharge letters (IDLs) for patients discharged from two functional old age psychiatry inpatient wards at the Royal Edinburgh Hospital. This patient group often have comorbid medical conditions and therefore monitoring of their psychotropic medication is especially important. The aim of the QI project was for 100% of patients discharged from thesewards on lithium or antipsychotic medication to have appropriate advice documented on their immediate discharge letter (IDL) with regards to medication monitoring.

**Method:**

Data were collected monthly by reviewing the notes of all discharged patients to determine the frequency at which medication monitoring advice was documented on IDLs from the two wards. A proposed new template for discharge letters which included advice on medication monitoring was discussed and agreed with the old age psychiatry team in Edinburgh. This was disseminated to the appropriate medical staff members and was included in induction packs for junior doctors. Following this a new “canned text” template was implemented to automatically populate the discharge letter with advice depending on whether they were antipsychotics/lithium/neither.

**Result:**

IDLs for 91 patients discharged between May 2020 and February 2021 were reviewed. Baseline data showed that 0% of patients (n = 15) had appropriate monitoring advice documented on their IDL. Following initial introduction of monitoring advice to the induction pack for junior doctors, the mean frequency of completed advice on IDLs was 50.9% across 6 months. Following implementation of the canned text, the frequency of completed advice on discharge letters for February 2021 was 100% (n = 7).

**Conclusion:**

This QI project has been successful in improving the rate of appropriate advice for antipsychotic and lithium monitoring being provided on immediate discharge letters. It is hoped that this will help reduce adverse effects associated with antipsychotics and lithium in older psychiatric patients. Further work could be done on determining the frequency that the advised monitoring is being carried out.

